# Patterns and characteristics of dyslipidemia subtypes among community-dwelling elderly in Eastern China: a cross-sectional study

**DOI:** 10.3389/fcvm.2025.1634134

**Published:** 2025-08-22

**Authors:** Xiaomeng Mi, Suting Xiong, Wenguo Xu, Fang Yao, Jie Huang, Lan Cui, Yu Qin, Jian Su, Wenchao Xu, Ran Tao, Jinyi Zhou

**Affiliations:** ^1^ Chronic Non-Communicable Disease Prevention and Control Section, Changzhou Center for Disease Control and Prevention, Changzhou Institute for Advanced Study of Public Health, Nanjing Medical University, Changzhou, China; ^2^ Chronic Non-Communicable Disease Prevention and Control Section, Nantong Center for Disease Control and Prevention, Nantong, China; ^3^ Chronic Non-Communicable Disease Prevention and Control Section, Jiangsu Center for Disease Control and Prevention, Nanjing, China

**Keywords:** dyslipidemia, dyslipidemia subtypes, dyslipidemia patterns, community-dwelling elderly Chinese, gender differences

## Abstract

**Background:**

Identifying and understanding different dyslipidemia patterns is crucial for maintaining the cardiovascular health of older adults. Therefore, this study aimed to investigate the dyslipidemia profiles of the elderly population from communities in an Eastern Chinese province, focusing on dyslipidemia subtypes and patterns, and exploring the associated demographic and health-related factors.

**Methods:**

A cross-sectional survey was conducted in communities in an Eastern Chinese province. Dyslipidemia patterns were defined using 4-digit binary codes for abnormal TC, TG, LDL-C, and HDL-C. Correspondence analysis explored subtype-pattern associations to reveal common combinations. Binary and multinomial logistic regressions, with Bonferroni correction, examined relationships between factors and dyslipidemia patterns.

**Results:**

Among 44,304 participants (31.5% dyslipidemia), correspondence analysis delineated patterns across Hyper_TC, Hyper_TG, Hyper_LDL, and Hypo_HDL subtypes, including Hyper_TC/LDL co-occurrence and Hyper_TG/Hypo_HDL independence, varying by gender. Multifactorial analyses revealed gender-based effects of age, education, income, and lifestyle, but consistent risks from comorbidities and urban-rural factors. Dyslipidemia subtype patterns and risk factor associations are thus gender- and pattern-specific.

**Conclusion:**

This study provided an in-depth analysis of dyslipidemia subtype patterns among community-dwelling elderly in Eastern China. The findings emphasized that considering gender- and pattern-specific risk factors is crucial in the prevention and management of dyslipidemia among older adults.

## Introduction

Dyslipidemia, involving abnormal levels of total cholesterol (TC), triglycerides (TG), low-density lipoprotein cholesterol (LDL-C), or high-density lipoprotein cholesterol (HDL-C), is a major global health issue linked to cardiovascular disease and other adverse outcomes like type 2 diabetes and non-alcoholic fatty liver disease (1–4). Multiple lipid abnormalities often coexist, amplifying health risks (5, 6).

Community-dwelling elderly individuals are particularly vulnerable due to age-related metabolic changes, comorbidities, and socioeconomic factors, especially in regions like China (4, 7–9). In contrast to older patients in hospitals who are often treated for sudden conditions, this community-based population could be in earlier stages of chronic disease management, offering a crucial window for timely treatments to control dyslipidemia and reduce heart risks, possibly leading to different patterns of lipid profiles (10, 11). Therefore, in-depth, pattern-based studies in this group of elderly people living in the community are important for creating specific management.

However, traditional dyslipidemia research focusing on single lipid subtypes (e.g., high TC or low HDL-C) overlooks the complexity of combined lipid abnormalities (12–14). While these subtype-specific studies have certainly contributed to our comprehension of single lipid abnormalities and their corresponding risk factors, they simultaneously possess limitations when considering combinations of lipid problems (13). Furthermore, the influence of risk factors like socioeconomic status (SES), lifestyle (smoking, alcohol), and gender often yields conflicting results in observational studies (15–18). There are differences in risk profiles of dyslipidemia between males and females (19, 20). The roles of smoking and alcohol are also complicated and not agreed upon (18, 21). These conflicting findings point to the requirement for more detailed approaches. Understanding which risk factors are linked to specific dyslipidemia patterns is crucial to developing more targeted ways to prevent and manage dyslipidemia.

To address these gaps, a pattern-based approach considering combinations of dyslipidemia subtypes is needed. This study utilizes correspondence analysis and multinomial logistic regression (22, 23) to analyze data from community-dwelling elderly (65–75 years) in Eastern China. Our aims are to: (1) identify prevalent dyslipidemia patterns; (2) determine demographic, socioeconomic, lifestyle, and health factors associated with these patterns; and (3) explore gender-specific differences. This pattern-focused analysis seeks to inform targeted dyslipidemia management strategies for this population.

## Methods

### Population and data collection

This study used data from the China PEACE-MPP (China Patient-centered Evaluative Assessment of Cardiac Events Million Persons Project), a large-scale cardiovascular disease screening program (2016–2023) (17, 24). Community-dwelling residents aged 35–75 years residing in project areas for ≥6 months were recruited via community mobilization. Written informed consent was obtained, and the Fuwai Hospital Ethics Committee approved the protocol. Data included face-to-face interviews using a standardized questionnaire (demographics, lifestyle, medical history), anthropometrics (height, weight for Body Mass Index calculation), and fasting finger-prick blood lipid measurements (TC, TG, LDL-C, HDL-C) using the CardioChek analyzer (a widely used and reliable analyser in epidemiological investigations) (25). This analysis includes 44,304 elderly individuals (65–75 years) from Jiangsu Province communities who completed examinations and interviews.

### Dyslipidemia subtypes and patterns

#### Subtypes of dyslipidemia

Dyslipidemia subtypes followed Chinese Guidelines (2023) (4):

Hyper_TC: TC levels ≥6.2 mmol/L.
Hyper_TG: TG levels ≥2.3 mmol/L.
Hyper_LDL: LDL-C levels ≥4.1 mmol/L.
Hypo_HDL: HDL-C levels <1.0 mmol/L.
Participants with ≥1 abnormality were considered dyslipidemic.

#### Patterns of dyslipidemia

Represented combinations of these abnormalities using a 4-digit binary code (TC, TG, LDL-C, HDL-C order; 1 = abnormal, 0 = normal). E.g., “1 0 0 1” indicates high TC and low HDL-C. Patterns were classified as Simple (1 abnormality), Mixed (2 abnormalities), or Complex (≥3 abnormalities).

### Characteristic variables

#### Demographics

Age (continuous), gender, residence (rural/urban), marital status (unmarried/married), annual family income (<50 k/≥50 k yuan), insurance (uninsured/insured), education (primary/below, junior high, senior high, college/above).

#### Health-related factors

BMI (body mass index) was categorized: Underweight/Normal weight (≤23.9 kg/m^2^), Overweight (24.0–27.9 kg/m^2^), and Obesity (≥28.0 kg/m^2^); Smoking status was categorized as never, <3 cigarettes/week, and ≥3 cigarettes/week. Alcohol consumption was classified into four groups: ≤1 time/month, 2–4 times/month, 2–3 times/week, and ≥4 times/week. Comorbidity number was defined as the total number of coexisting chronic diseases. Participants were asked if they had a diagnosis of any of these chronic conditions: hypertension, diabetes, angina pectoris, myocardial infarction, stroke, chronic obstructive pulmonary disease, kidney or ureteral stones, and cancer.

### Statistical analysis

Analyses used R 4.3.2. Descriptive statistics summarized participant characteristics by dyslipidemia subtype [mean ± SD or median [IQR] for continuous; *n* (%) for categorical; Chi-square tests for group differences]. To address missing data, multiple imputation by chained equations (MICE) was utilized. Correspondence analysis (CA) explored associations between dyslipidemia subtypes and patterns (22). Binary logistic regression assessed factors associated with overall dyslipidemia (any abnormality). Multinomial logistic regression (MLR) identified factors associated with specific dyslipidemia patterns (reference: no dyslipidemia) (23). Odds ratios (ORs) with 95% Confidence Intervals (CIs) were calculated. MLR *P*-values were Bonferroni-adjusted. Forest plots visualized associations (protective green, risk red). Results for patterns with *n* < 100 (“Other patterns”) are in Supplementary Figure and considered exploratory. Gender-stratified subgroup analyses were performed for CA and logistic regressions.

Odds ratios (ORs) and their corresponding 95% confidence intervals (CIs) were calculated to quantify the associations between variables and both overall dyslipidemia and the specific dyslipidemia patterns. To control for the family-wise Type I error rate arising from multiple comparisons, *P*-values generated from the MLR were adjusted using the Bonferroni correction method. Forest plots were generated for all patterns to visually represent these associations, clearly indicating protective factors in green and risk factors in red. Results for less prevalent patterns (*n* < 100), which were grouped as “Other patterns”, are presented in the Supplementary Figure. Logistic regression models require a minimum of approximately 10 events per predictor variable to ensure stable parameter estimates and adequate statistical power (23). Due to the limited sample size, these results should be considered exploratory.

### Subgroup analysis

We performed gender-based analyses for both correspondence analysis and two types of logistic regressions to examine gender-specific risk factors associated with dyslipidemia patterns. These analyses allowed us to explore the influence of dyslipidemia subtypes, demographic characteristics, and health status within each gender.

## Results

### Characteristics of individuals included

Among 44,304 participants, 13,960 (31.5%) had dyslipidemia. Prevalence rates for subtypes were: Hyper_TC 7.2%, Hyper_TG 14.6%, Hyper_LDL 4.1%, and Hypo_HDL 16.5%. Table 1 details participant characteristics stratified by subtype. Females were more prevalent in Hyper_TC, Hyper_TG, and Hyper_LDL groups, while males predominated in the Hypo_HDL group. Significant differences (*P* < 0.05) between dyslipidemic and non-dyslipidemic groups were observed for residence, income, education, smoking, alcohol use, BMI level, and comorbidity number, but not insurance status. Lipid levels (TC, TG, LDL-C, HDL-C) and BMI also differed significantly across subtypes.

**Table 1:** Characteristics of demographic and health-related factors Among elderly population included.

Variables	Total (*N*, %) 44,304 (100)	Dyslipidemia (*n*, %) 13,960 (31.5)	*P*_1_	Hyper_TC (*n*, %) 3,177 (7.2)	*P*_2_	Hyper_TG (*n*, %) 6,481 (14.6)	*P*_3_	Hyper_LDL (*n*, %) 1,834 (4.1)	*P*_4_	Hypo_HDL (*n*, %) 7,316 (16.5)	*P*_5_
Age (years)	69.00 [66.00, 71.00]	69.00 [66.00, 71.00]	0.76	69.00 [67.00, 71.00]	0.44	68.00 [66.00, 71.00]	<0.05	69.00 [67.00, 71.00]	0.58	69.00 [66.00, 71.00]	0.44
Gender (%)			0.49		<0.05		<0.05		<0.05		<0.05
Male	20,293 (45.8)	6,360 (45.6)		751 (23.6)		2,251 (34.7)		545 (29.7)		4,594 (62.8)	
Female	24,011 (54.2)	7,600 (54.4)		2,426 (76.4)		4,230 (65.3)		1,289 (70.3)		2,722 (37.2)	
Area (%)			<0.05		<0.05		<0.05		<0.05		<0.05
Rural	21,547 (48.6)	5,983 (42.9)		1,361 (42.8)		2,810 (43.4)		690 (37.6)		3,018 (41.3)	
Urban	22,757 (51.4)	7,977 (57.1)		1,816 (57.2)		3,671 (56.6)		1,144 (62.4)		4,298 (58.7)	
Marital_status (%)			<0.05		<0.05		0.46		0.50		<0.05
Unmarried	4,861 (11.0)	1,423 (10.2)		398 (12.5)		728 (11.2)		209 (11.4)		608 (8.3)	
Married	39,443 (89.0)	12,537 (89.8)		2,779 (87.5)		5,753 (88.8)		1,625 (88.6)		6,708 (91.7)	
Income (%)			0.08		<0.05		0.68		0.05		<0.05
<50, 000 yuan	34,861 (78.7)	10,914 (78.2)		2,618 (82.4)		5,092 (78.6)		1,477 (80.5)		5,557 (76.0)	
≥50,000 yuan	9,443 (21.3)	3,046 (21.8)		559 (17.6)		1,389 (21.4)		357 (19.5)		1,759 (24.0)	
Insurance (%)			0.29		0.63		0.72		0.86		0.52
Uninsured	595 (1.3)	175 (1.3)		39 (1.2)		84 (1.3)		24 (1.3)		92 (1.3)	
Insured	43,709 (98.7)	13,785 (98.7)		3,138 (98.8)		6,397 (98.7)		1,810 (98.7)		7,224 (98.7)	
Education (%)			<0.05		<0.05		0.41		<0.05		<0.05
Primary school and below	31,763 (71.9)	9,586 (68.9)		2,474 (78.0)		4,625 (71.5)		1,354 (73.9)		4,568 (62.7)	
Junior high school	8,886 (20.1)	3,000 (21.6)		507 (16.0)		1,294 (20.0)		322 (17.6)		1,837 (25.2)	
Senior high school	2,753 (6.2)	1,025 (7.4)		158 (5.0)		435 (6.7)		122 (6.7)		653 (9.0)	
College degree and above	761 (1.7)	310 (2.2)		34 (1.1)		111 (1.7)		35 (1.9)		230 (3.2)	
Smoking (%)			0.013		<0.05		<0.05		<0.05		<0.05
Never	34,701 (78.3)	11,049 (79.1)		2,768 (87.1)		5,354 (82.6)		1,551 (84.6)		5,331 (72.9)	
<3 cigarettes/week	1,838 (4.1)	543 (3.9)		90 (2.8)		202 (3.1)		53 (2.9)		364 (5.0)	
≥3 cigarettes/week	7,765 (17.5)	2,368 (17.0)		319 (10.0)		925 (14.3)		230 (12.5)		1,621 (22.2)	
Alcohol (%)			<0.05		<0.05		<0.05		<0.05		<0.05
≤1 time/month	34,110 (77.3)	1,1,083 (79.7)		2,657 (83.9)		5,218 (80.7)		1,489 (81.4)		5,651 (77.7)	
2–4 times/month	1,867 (4.2)	638 (4.6)		112 (3.5)		284 (4.4)		85 (4.6)		394 (5.4)	
2–3 times/week	1,453 (3.3)	435 (3.1)		63 (2.0)		165 (2.6)		51 (2.8)		282 (3.9)	
≥4 times/week	6,676 (15.1)	1,747 (12.6)		334 (10.5)		800 (12.4)		205 (11.2)		949 (13.0)	
BMI_Level (%)			<0.05		0.09		<0.05		<0.05		<0.05
≤23.9 kg/m^2^	16,415 (37.2)	3,670 (26.3)		1,120 (35.3)		1,456 (22.5)		603 (33.0)		1,691 (23.2)	
24.0–27.9 kg/m^2^	18,754 (42.4)	6,534 (46.9)		1,379 (43.5)		3,105 (48.0)		845 (46.2)		3,545 (48.6)	
≥28.0 kg/m^2^	9,014 (20.4)	3,727 (26.8)		670 (21.1)		1,907 (29.5)		382 (20.9)		2,064 (28.3)	
Comorbidity number (%)			<0.05		<0.05		<0.05		<0.05		<0.05
0	9,870 (22.3)	2,242 (16.1)		480 (15.1)		846 (13.1)		269 (14.7)		1,233 (16.9)	
1	23,256 (52.5)	7,130 (51.1)		1,703 (53.6)		3,231 (49.9)		975 (53.2)		3,659 (50.0)	
2	8,700 (19.6)	3,578 (25.6)		791 (24.9)		1,973 (30.4)		460 (25.1)		1,834 (25.1)	
3	1,448 (3.3)	541 (3.9)		110 (3.5)		218 (3.4)		69 (3.8)		320 (4.4)	
4	814 (1.8)	344 (2.5)		80 (2.5)		159 (2.5)		56 (3.1)		185 (2.5)	
5	179 (0.4)	100 (0.7)		12 (0.4)		42 (0.6)		5 (0.3)		67 (0.9)	
6	33 (0.1)	21 (0.2)		1 (0.0)		11 (0.2)		0 (0.0)		15 (0.2)	
7	4 (0.0)	4 (0.0)		0 (0.0)		1 (0.0)		0 (0.0)		3 (0.0)	
BMI (kg/m^2^)	24.97 [22.82, 27.23]	25.81 [23.78, 27.97]	<0.05	25.07 [22.98, 27.32]	<0.05	26.14 [24.09, 28.25]	<0.05	25.08 [23.31, 27.33]	<0.05	26.08 [24.06, 28.11]	<0.05
TC (mmol/L)	4.53 [3.87, 5.23]	4.73 [3.78, 6.06]	<0.05	6.70 [6.41, 7.25]	<0.05	5.03 [4.31, 5.85]	<0.05	6.73 [6.28, 7.29]	<0.05	3.94 [3.34, 4.64]	<0.05
TG(mmol/L)	1.30 [0.94, 1.86]	2.16 [1.35, 2.91]	<0.05	1.86 [1.30, 2.75]	<0.05	2.97 [2.56, 3.70]	<0.05	1.65 [1.22, 2.32]	<0.05	1.67 [1.17, 2.46]	<0.05
LDL (mmol/L)	2.43 [1.89, 3.02]	2.55 [1.88, 3.51]	<0.05	4.13 [3.69, 4.58]	<0.05	2.47 [1.81, 3.21]	<0.05	4.54 [4.29, 4.99]	<0.05	2.21 [1.69, 2.78]	<0.05
HDL (mmol/L)	1.37 [1.13, 1.67]	1.03 [0.92, 1.37]	<0.05	1.60 [1.31, 1.94]	<0.05	1.17 [0.98, 1.40]	<0.05	1.44 [1.19, 1.71]	<0.05	0.93 [0.84, 0.99]	<0.05

Hyper_TC, total cholesterol (TC) levels ≥6.2 mmol/L; Hyper_TG, triglycerides (TG) levels ≥2.3 mmol/L; Hyper_LDL, low-density lipoprotein cholesterol (LDL-C) levels ≥4.1 mmol/L; Hypo_HDL, high-density lipoprotein cholesterol (HDL-C) levels <1.0 mmol/L. Data are presented as median [interquartile range] for age, BMI, TC, TG, LDL, and HDL. And *n* (percentage) for categorical variables. *P*-values were calculated using the Kruskal–Wallis test for age, BMI, TC, TG, LDL-C, and HDL-C, and the Chi-square test for categorical variables.

*P*1, Comparison of the Dyslipidemia group with its negative control group. *P*2, Comparison of the Hyper_TC group with its negative control group. *P*3, Comparison of the Hyper_TG group with its negative control group. *P*4, Comparison of the Hyper_LDL group with its negative control group. *P*5: Comparison of the Hypo_HDL group with its negative control group.

### Dyslipidemia subtypes & patterns

Table 2 shows pattern distributions. The most common overall patterns were “0 0 0 1” (Hypo_HDL, 36.5%) and “0 1 0 0” (Hyper_TG, 24.6%), followed by “0 1 0 1” (Hyper_TG & Hypo_HDL, 13.2%). Major patterns differed by gender: “0001” (Hypo_HDL) was most common in males (54.6%), while “0 1 0 0” (Hyper_TG) led in females (31.9%). Females had proportionally more Hyper_TC patterns (“1 X X X”), whereas males had a substantially higher proportion of the “0 0 0 1” pattern.

**Table 2:** Distribution of dyslipidemia patterns.

Dyslipidemia Patterns		*N* (Composition %)^a^		
		Overall	Males	Females
Single Abnormality	0 0 0 1	5,094 (36.5)	3,474 (54.6)	1,620 (21.3)
	0 1 0 0	3,431 (24.6)	1,006 (15.8)	2,425 (31.9)
	1 0 0 0	963 (6.9)	201 (3.2)	762 (10.0)
	0 0 1 0	233 (1.7)	93 (1.5)	140 (1.8)
Mixed Abnormality	0 1 0 1	1,849 (13.2)	946 (14.9)	903 (11.9)
	1 0 1 0	1,025 (7.3)	257 (4.0)	768 (10.1)
	1 1 0 0	664 (4.8)	140 (2.2)	524 (6.9)
	0 0 1 1	78 (0.6)	46 (0.7)	32 (0.4)
	1 0 0 1	49 (0.4)	21 (0.3)	28 (0.4)
	0 1 1 0	39 (0.3)	12 (0.2)	27 (0.4)
Complex Abnormality	1 1 1 0	289 (2.1)	57 (0.9)	232 (3.1)
	1 1 0 1	76 (0.5)	27 (0.4)	49 (0.6)
	0 1 1 1	59 (0.4)	32 (0.5)	27 (0.4)
	1 0 1 1	37 (0.3)	17 (0.3)	20 (0.3)
	1 1 1 1	74 (0.5)	31 (0.5)	43 (0.6)
Total		13,960 (100)	6,360 (100)	7,600 (100)

^a^ These three columns in the table show the sample size and subtype composition of dyslipidemia patterns in the total population, males, and females, respectively. The columns add up to 100%.

Dyslipidemia patterns are defined by a 4-digit binary code representing the presence (1) or absence (0) of abnormal levels of total cholesterol (TC), triglycerides (TG), low-density lipoprotein cholesterol (LDL-C), and high-density lipoprotein cholesterol (HDL-C), in that order. Dyslipidemia patterns were classified into three levels: Simple (isolated abnormalities in TC, TG, LDL-C, and HDL-C), Mixed (combinations of two abnormalities), and Complex (three or more concurrent abnormalities).

**Figure 1:**
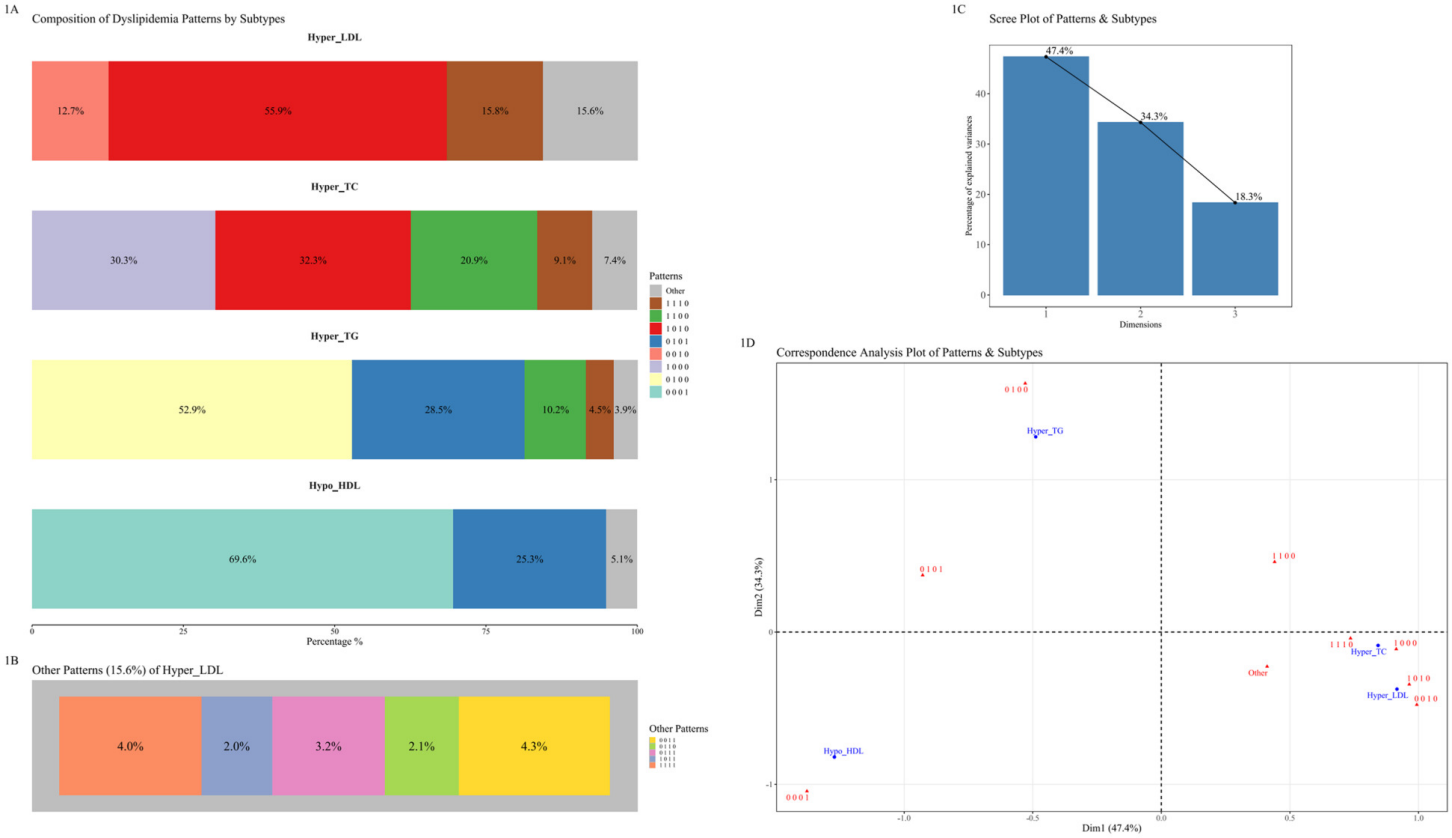
Correspondence analysis of dyslipidemia subtypes and patterns. **(A)** Composition of dyslipidemia patterns for each of the four main subtypes. Each bar illustrates the percentage distribution of different patterns within Hyper_LDL*,* Hyper_TC, Hyper_TG*,* and Hypo*_*HDL subtypes. **(B)** A detailed breakdown of the “Other Patterns” category for the Hyper_LDL subtype. **(C)** Scree plot showing the percentage of inertia (variance) explained by the first two dimensions of the correspondence analysis. **(D)** Correspondence analysis plot illustrating the associations between dyslipidemia subtypes (in red) and specific patterns (in blue). Proximity between points indicates a stronger association.

Correspondence analysis (Figure 1) revealed pattern structures. Hypo_HDL and Hyper_TG contributed most to simple patterns (Figure 1A). Dimensions 1 and 2 explained >80% of the association (Figure 1C). Hyper_TC and Hyper_LDL clustered together, strongly associated with mixed and complex patterns (Figure 1D). Hyper_LDL, in particular, showed low contribution to simple patterns (<15%) and higher contribution to complex ones, suggesting more complex metabolic dysregulation when LDL-C is high (Figures 1A,B). Gender differences were observed: males showed stronger tendencies towards “0001” and complex patterns involving Hyper_LDL or Hyper_TC compared to females (detailed breakdown in Supplementary Figures S1.1, S1.2).

### Logistic regression analysis (overall dyslipidemia)

Neither age nor gender was associated with overall dyslipidemia risk in this 65–75 age group (ORs = 1.00, 0.96; *P* > 0.05). Insurance and income were also not associated with overall dyslipidemia. However, being married, urban residence, higher education (junior high+), frequent smoking (≥3/week), higher BMI (≥24.0 kg/m^2^), and more comorbidities were significantly associated with higher odds of dyslipidemia (*P* < 0.05), while frequent alcohol consumption was protective (Supplementary Figure S2). Gender-specific differences emerged: age and frequent alcohol use conferred protection only in males; higher income was associated with increased dyslipidemia odds in males but with reduced odds in females. Moreover, higher education elevated dyslipidemia odds solely in males, and being married raised the odds exclusively in females (Supplementary Figures S3.1, S3.2).

### Multinomial logistic regression analysis (specific dyslipidemia patterns)

**Figure 2:**
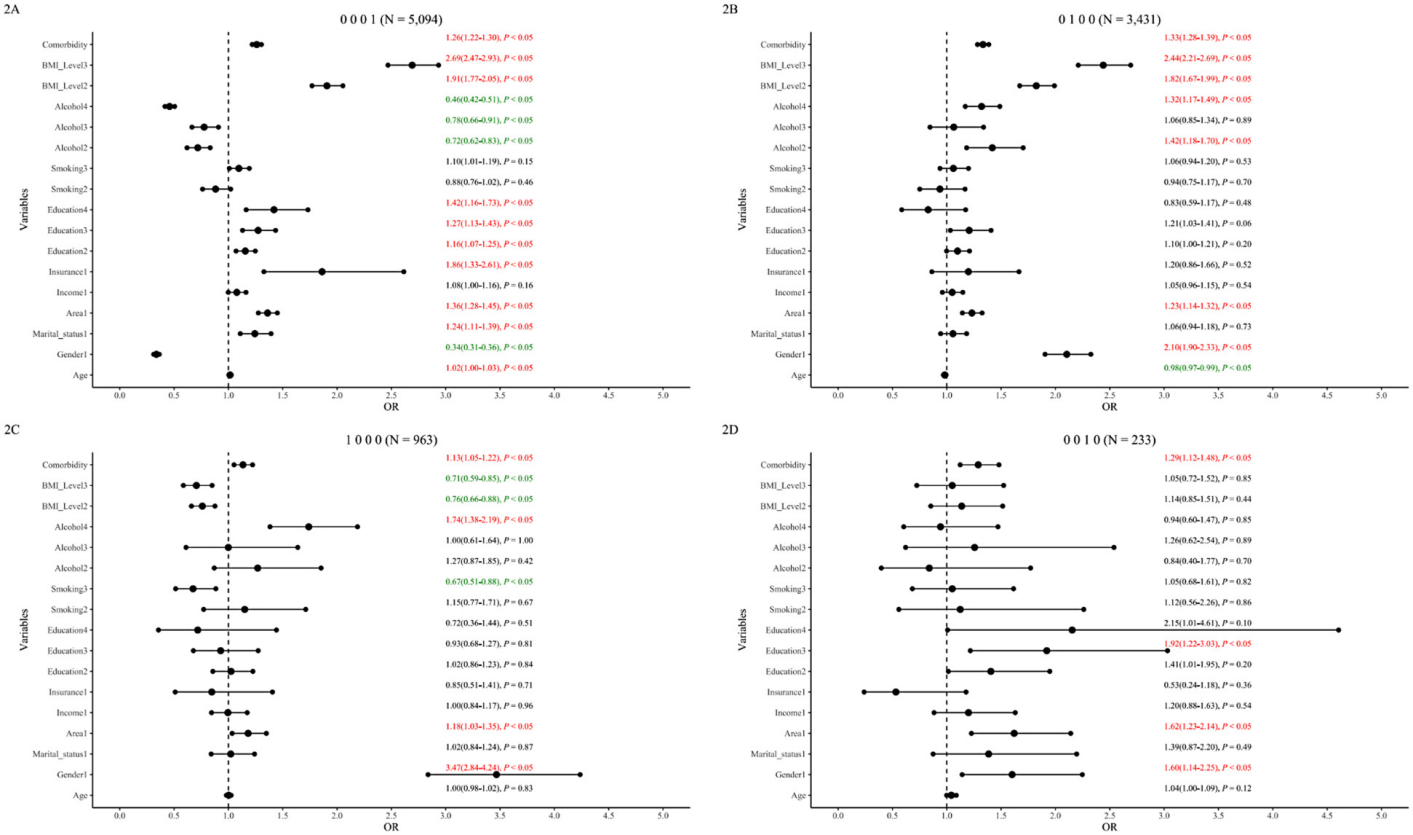
Single dyslipidemia patterns and associated factors in the 65–75 age group. The figure displays forest plots for the four most prevalent single dyslipidemia patterns: **(A)** isolated hypo_HDL (“0001”), **(B)** Isolated Hyper_TG (“0100”), **(C)** isolated hyper_TC (“1000”), and **(D)** isolated hyper_LDL (“0010”). Odds ratios (ORs) with 95% confidence intervals (CIs) are shown. For clarity, factors associated with lower odds are represented in green, and factors associated with higher odds are in red.

**Figure 3:**
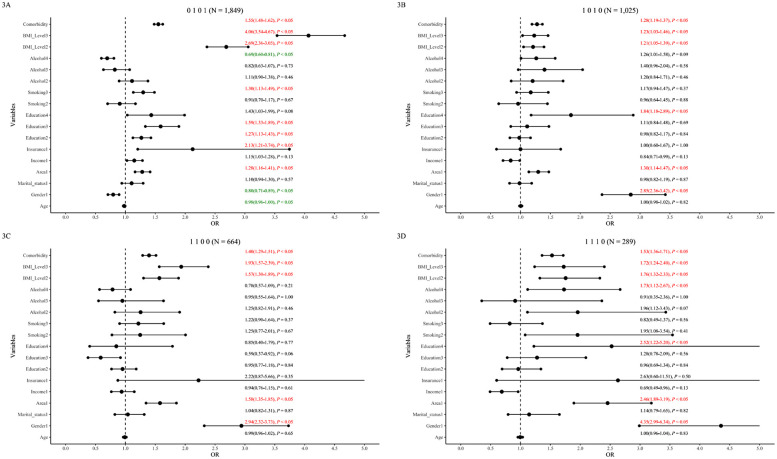
Mixed & Complex dyslipidemia patterns and associated factors in the 65–75 age group. The figure displays forest plots for prevalent mixed and complex dyslipidemia patterns: **(A)** hyper_TG & hypo*_*HDL (“0101”), **(B)** hyper*_*TC & hyper*_*LDL (“1010”), **(C)** hyper*_*TC & hyper*_*TG (“1100”), and **(D)** hyper*_*TC*&* hyper*_*TG & hyper_LDL (“1110”). Odds ratios (ORs) with 95% confidence intervals (CIs) are shown. For clarity, factors associated with lower odds are represented in green, and factors associated with higher odds are in red.

Associations for the eight most prevalent patterns are shown in Figures 2, 3 (less prevalent patterns in Supplementary Figures S4, S5).

#### Demographics

Age was associated with lower odds of TG-related patterns (“0 1 0 0”, “0 1 0 1”) overall and significantly so in males (Supplementary Figures S4.1, S5.1). High income was not significantly associated overall but was associated with higher odds of “0001” in males and showed a protective trend for “1 0 1 0” in females (*P* = 0.07 adjusted). Insurance status was associated with higher odds of “0 0 0 1” and “0 1 0 1” overall but not significantly associated in gender subgroups. Urban residence was consistently associated with higher odds across most patterns in overall and male analyses, but not associated with “1 0 0 0” and “1 0 1 0” in females. Higher education was associated with dose-dependently higher odds for Hypo_HDL patterns (“0 0 0 1”, “0 1 0 1”) overall, significant only in males.

#### Health-related factors

Frequent smoking was associated with higher odds of “0 1 0 1” and lower odds of “1 0 0 0” overall. Gender-stratified: associated with higher odds of “0 0 0 1” and “0 1 0 1” in males, and lower odds of “1 0 0 0” in females. Frequent alcohol use showed complex associations: associated with higher odds for TG/TC patterns (“0 1 0 0”, “1 0 0 0”, “1 1 1 0”) but lower odds for Hypo_HDL patterns (“0 0 0 1”, “0 1 0 1”) overall and similarly in males; no significant associations in females. Overweight/obesity was generally associated with incrementally higher odds for most patterns but lower odds for “1 0 0 0” (overall and females). Comorbidities were associated with higher odds across patterns, though significance varied slightly by pattern and gender (Further details: Figures 2, 3; Supplementary Figures 4, 5).

## Discussion

Our study confirms a high dyslipidemia prevalence (31.5%) among community-dwelling older Chinese adults, emphasizing the need for pattern-specific risk analysis. The Hypo_HDL pattern (“0 0 0 1”) was most common overall and particularly in males, whereas the Hyper_TG pattern (“0 1 0 0”) predominated in females. These stark gender differences, alongside findings that urban residence and higher education were associated with higher odds of dyslipidemia, underscore the complex interplay of biological, socioeconomic, and lifestyle factors in shaping dyslipidemia profiles in this population.

### Gender dimorphism in dyslipidemia profiles: subtype and pattern differences

Marked gender dimorphism was evident. Females showed higher prevalence of patterns involving elevated TC, TG, and LDL-C, potentially linked to post-menopausal hormonal changes (16, 19, 26), while males predominantly exhibited Hypo_HDL patterns, possibly related to androgens or lifestyle factors like historically higher smoking rates and lower healthcare engagement (27–29). Interestingly, overweight/obesity paradoxically protected against the isolated Hyper_TC (“1 0 0 0”) pattern, an effect primarily seen in females, perhaps reflecting metabolically healthy obesity phenotypes or associated higher HDL_C levels (30).

### Lifestyle paradoxes: different impacts of alcohol on dyslipidemia

Lifestyle factors showed complex associations. Frequent alcohol consumption demonstrated a dual association, particularly in males, being associated with lower odds of Hypo_HDL related patterns (“0 0 0 1”, “0 1 0 1”) but associated with higher odds for others involving Hyper_TG or Hyper_TC (“0 1 0 0”, “1 0 0 0”, “1 1 1 0”). No protective association was observed in females; instead, less frequent drinking was associated with the “1 0 1 0” pattern. The two-way link between alcohol and dyslipidemia is well-known (17, 18). Drinking alcohol in normal amounts is often related to higher HDL-C (“good” cholesterol), but drinking too much could lead to higher triglycerides and other lipid problems (21). Literature suggests alcohol elevates HDL-C through pathways like stimulating ApoA-I synthesis, enhancing reverse cholesterol transport, inhibiting CETP, and augmenting lipoprotein lipase activity (31). While these mechanisms explain HDL's functionality and its response to moderate alcohol, the overall benefit of HDL-C remains debated (32). Given alcohol's known health risks, our finding is an epidemiological observation, not a public health recommendation.

### Socioeconomic paradoxes: wealth and dyslipidemia in elderly population

This study, conducted in Jiangsu Province –a province in China with a GDP per capita exceeding $20,000 USD –revealed notable associations between socioeconomic status and dyslipidemia in this elderly Chinese population. Socioeconomic status also revealed paradoxes. Urban living and higher education were associated with higher odds of dyslipidemia, especially in males (“0 0 0 1”, “0 1 0 1”), possibly reflecting adverse lifestyle shifts (diet, activity) accompanying urbanization despite potentially better healthcare access (8, 33–35). A notable observation was the elevated odds of low HDL-C (Hypo_HDL, pattern “0 0 0 1”) among men with higher education, likely stemming from increased sedentary behavior. This is supported by researches indicating that highly educated individuals tend to engage more frequently in sedentary activities, such as reading (36, 37). While occupational type was not collected in our dataset, this unmeasured factor could potentially mediate the observed association. Annual family income showed opposing associations by gender: higher income was associated with higher odds for the male-dominant “0 0 0 1” pattern but was associated with lower odds for the “1 0 1 0” pattern in females. These disparities might stem from gender differences in health prioritization and healthcare utilization influenced by economic standing. A research conducted in Guangdong Province, China, (economically strong province) has shown that individuals with higher incomes prioritize their health, especially in accessing chronic disease healthcare (38). And females' greater willingness to seek healthcare, compared to men, might be one contributing factor to this difference. These complicated associations, observed in elderly residents of a high-GDP Chinese province, highlighted the critical need to consider the specific socioeconomic and cultural context when assessing dyslipidemia risk in the elderly.

Key limitations include: First, the cross-sectional design, preventing causal inference, and the focus on a specific elderly (65–75 years) Chinese population, which may limit generalizability to other demographics. Second, POCT devices (the CardioChek analyzer) offer advantages for field screening, their accuracy/precision may differ somewhat from standard venous blood laboratory testing methods (25). Third, our “comorbidity number” variable relied on self-reported disease history, which is susceptible to recall bias and potential under-reporting.

The value of this research lies in revealing novel health paradoxes and association patterns that may emerge in regions at the forefront of economic transition and development, rather than aiming to provide generalizable conclusions for the entirety of East China. Our findings offer important insights into the health characteristics of economically advanced regions in China and lay the groundwork for future inter-regional comparative studies across a broader and more diverse East China.

## Conclusion

This study provided an in-depth analysis of dyslipidemia subtype patterns among elderly Chinese individuals in community settings. The findings emphasized that considering gender- and pattern-specific risk factors is crucial in the prevention and management of dyslipidemia among older adults.

## Data Availability

The raw data supporting the conclusions of this article will be made available by the authors, without undue reservation.
