# Supplementary material for: Patterns and characteristics of dyslipidemia subtypes among community-dwelling elderly in Eastern China: a cross-sectional study
Source: Front Cardiovasc Med. 2025 Aug 22;12:1634134. doi: 10.3389/fcvm.2025.1634134 (PMC12411541; doi:10.3389/fcvm.2025.1634134)

Supplementary Materials Legend

Supplemental Figure.1.1 Correspondence analysis histogram of Patterns&Subtypes among Males.

Supplemental Figure.1.2 Correspondence analysis histogram of Patterns&Subtypes among Females.

Supplemental Figure.2 Forest plot: Overall Dyslipidemia and Associated Factors in the 65-75 Age Group.

Supplemental Figure.3.1 Forest plot: Overall Dyslipidemia and Associated Factors in the 65-75 Age Group among Males.

Supplemental Figure.3.2 Forest plot: Overall Dyslipidemia and Associated Factors in the 65-75 Age Group among Females.

Supplemental Figure.4.1 Forest plot: Single Dyslipidemia Patterns and Associated Factors in the 65-75 Age Group among Males.

Supplemental Figure.4.2 Forest plot: Single Dyslipidemia Patterns and Associated Factors in the 65-75 Age Group among Females.

Supplemental Figure.5.1 Forest plot: Mixed & Complex Dyslipidemia Patterns and Associated Factors in the 65-75 Age Group among Males.

Supplemental Figure.5.2 Forest plot: Single Dyslipidemia Patterns and Associated Factors in the 65-75 Age Group among Females.

Supplemental Figure.1.1A

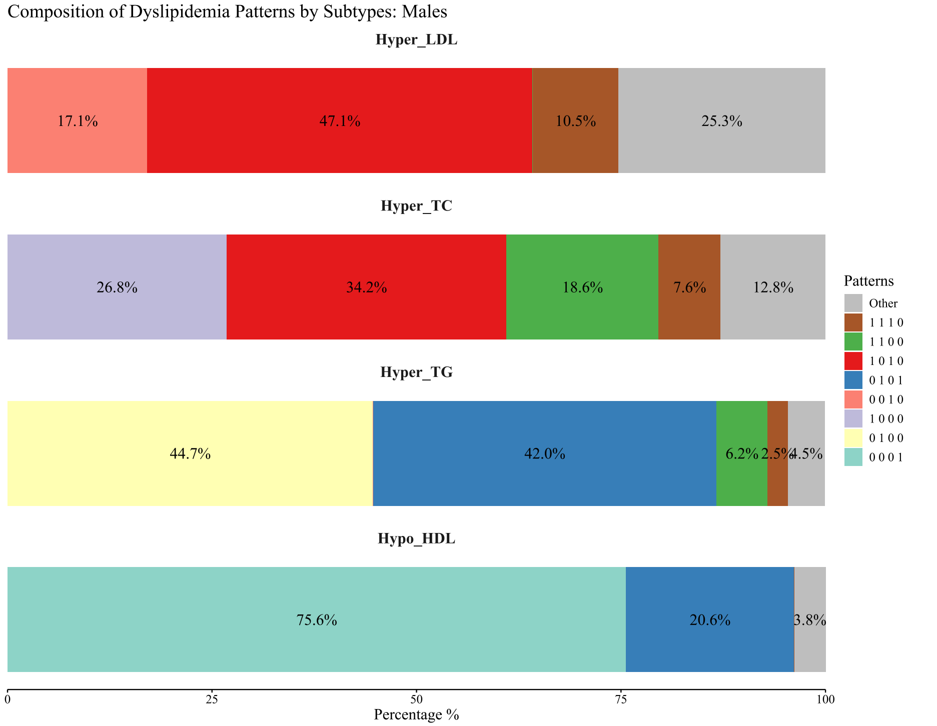

Supplemental Figure.1.1B

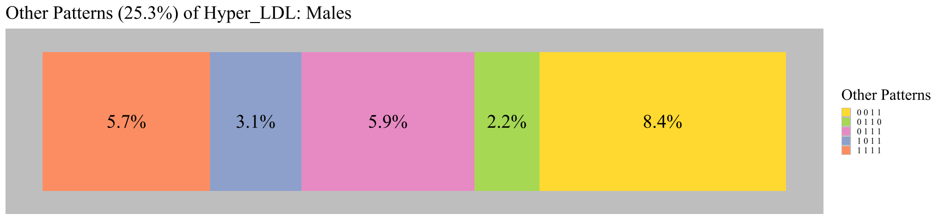

Supplemental Figure.1.1C

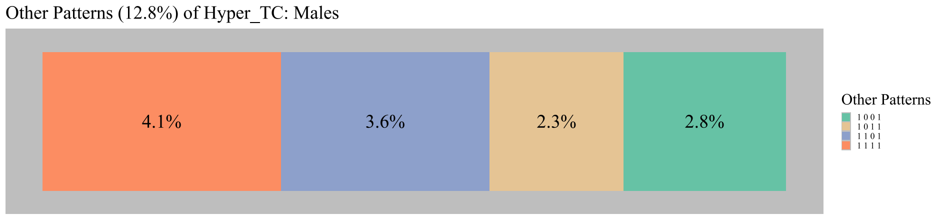

Supplemental Figure.1.1D

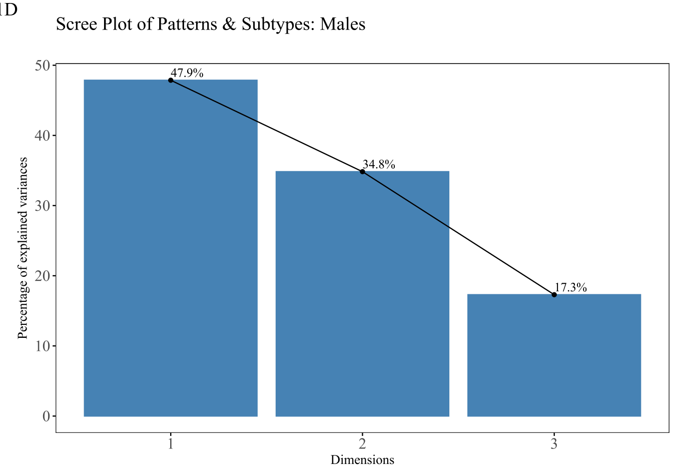

Supplemental Figure.1.1E

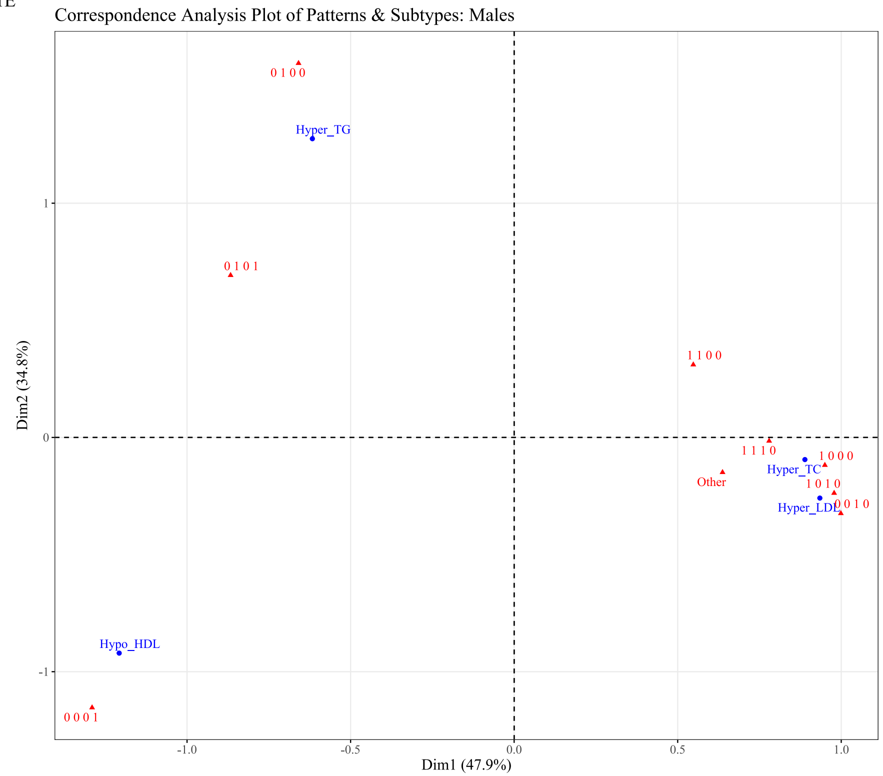

Supplemental Figure.1.2A

Composition of Dyslipidemia Patterns by Subtypes: Females

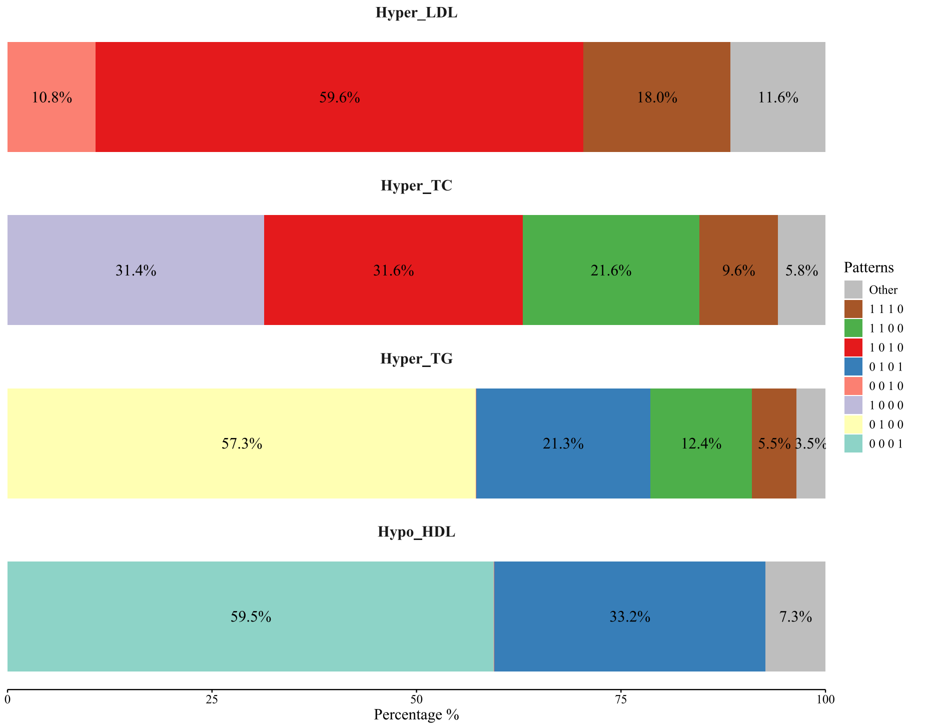

Supplemental Figure.1.2B

Other Patterns (11.6%) of Hyper_LDL: Females

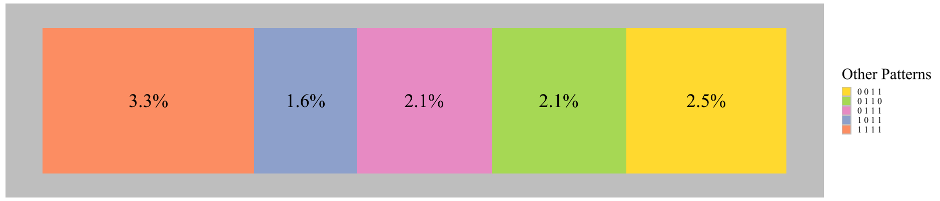

Supplemental Figure.1.2C

Scree Plot of Patterns & Subtypes: Females

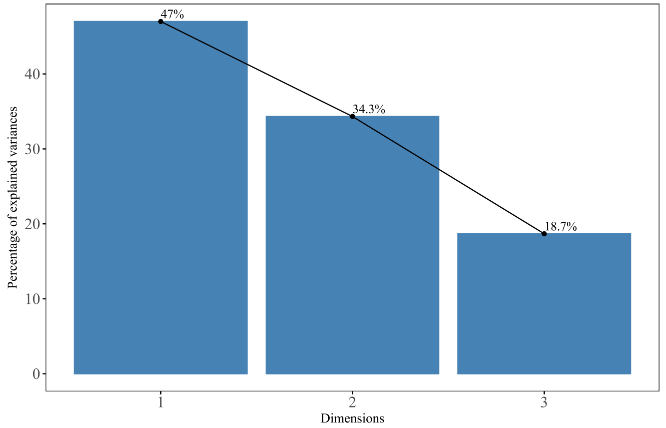

Supplemental Figure.1.2D

Correspondence Analysis Plot of Patterns & Subtypes: Females

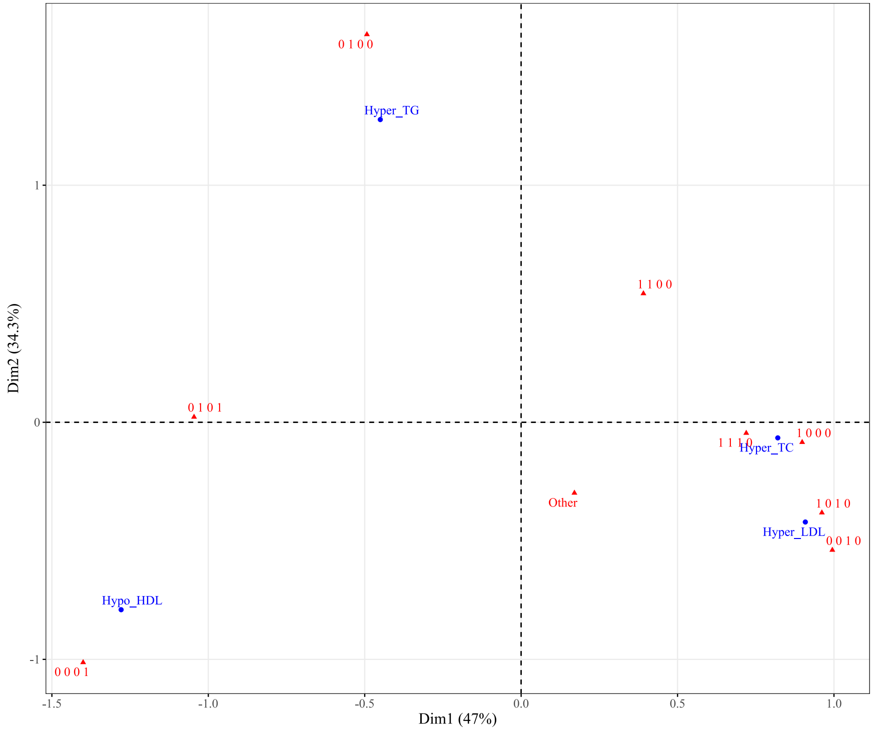

Supplemental Figure.2 Forestplot: Overall Dyslipidemia and Associated Factors in the 65-75 Age Group

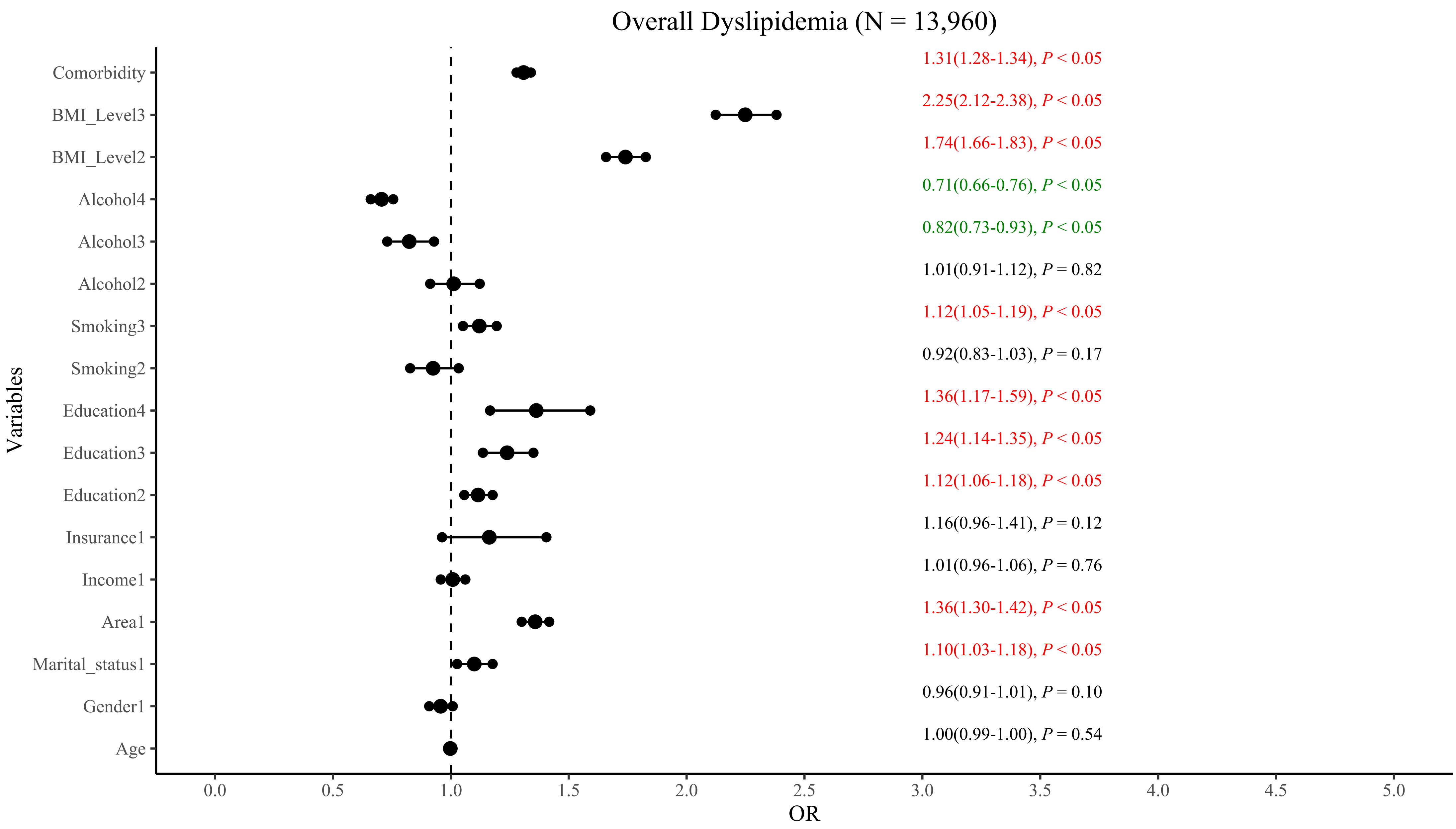

Supplemental Figure.3.1 Overall Dyslipidemia and Associated Factors among Males

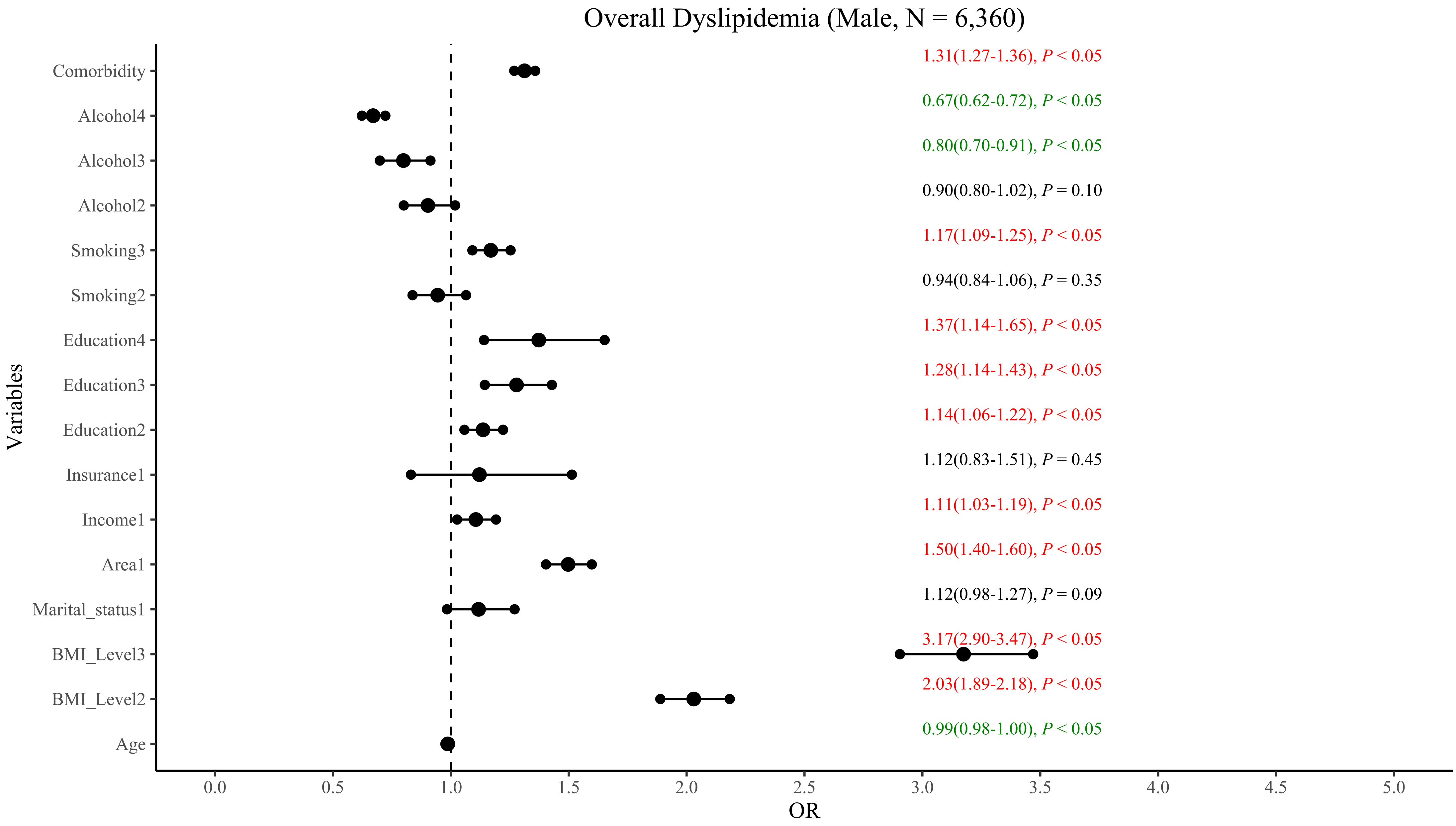

Supplemental Figure.3.2 Overall Dyslipidemia and Associated Factors among Females

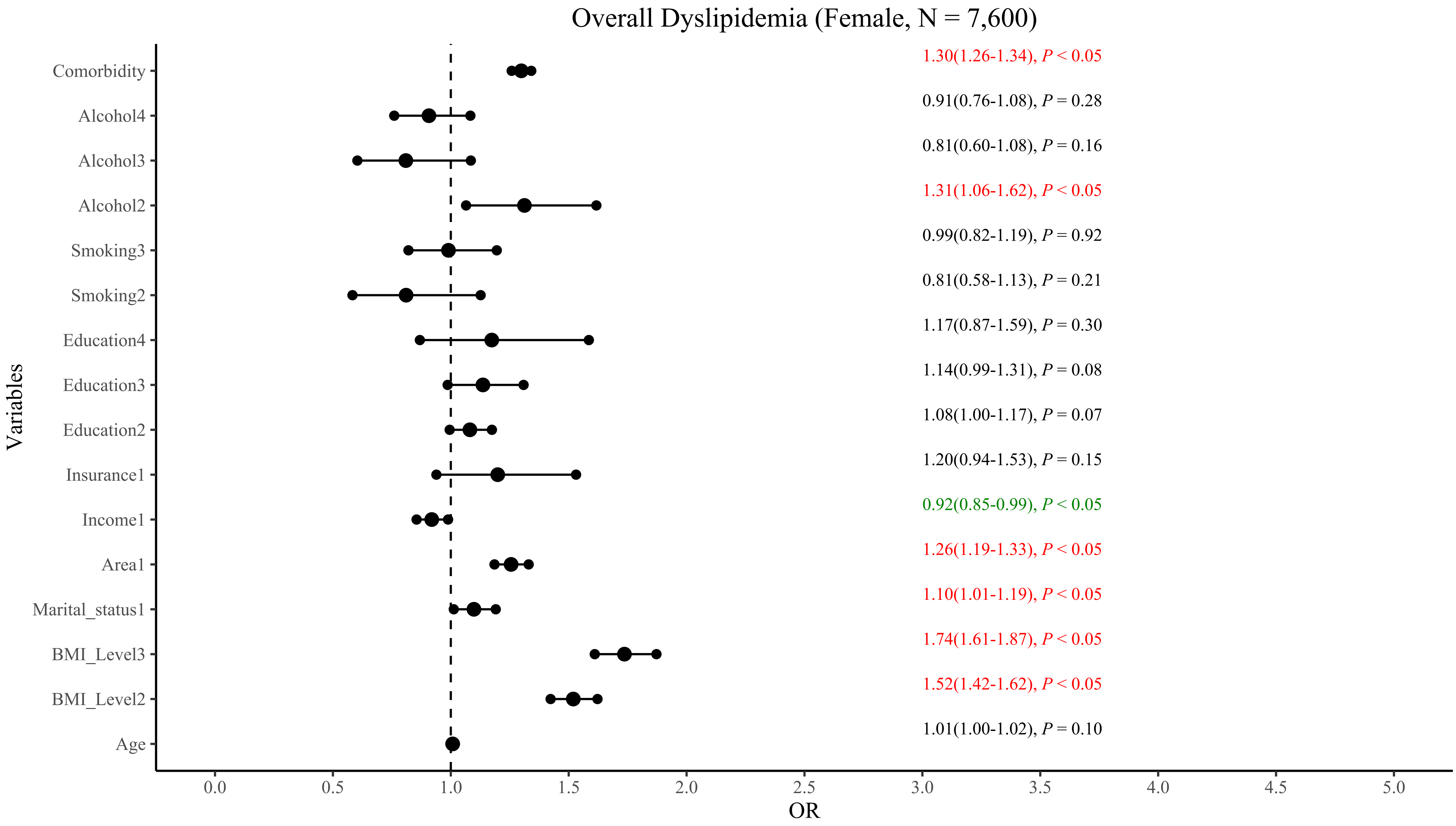

Supplemental Figure.4.1 Single Dyslipidemia Patterns and Associated Factors among Males

Supplemental Figure.4.1A

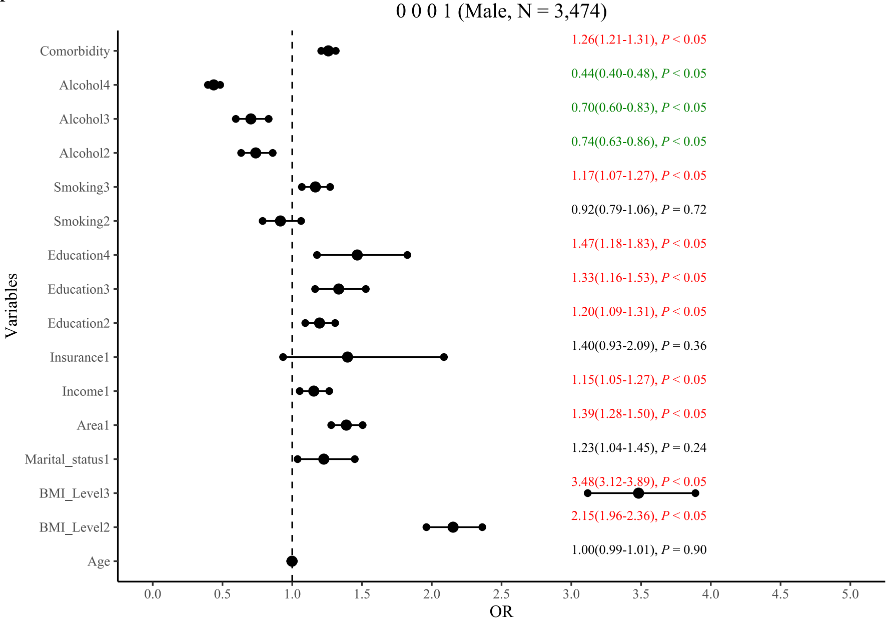

Supplemental Figure.4.1B

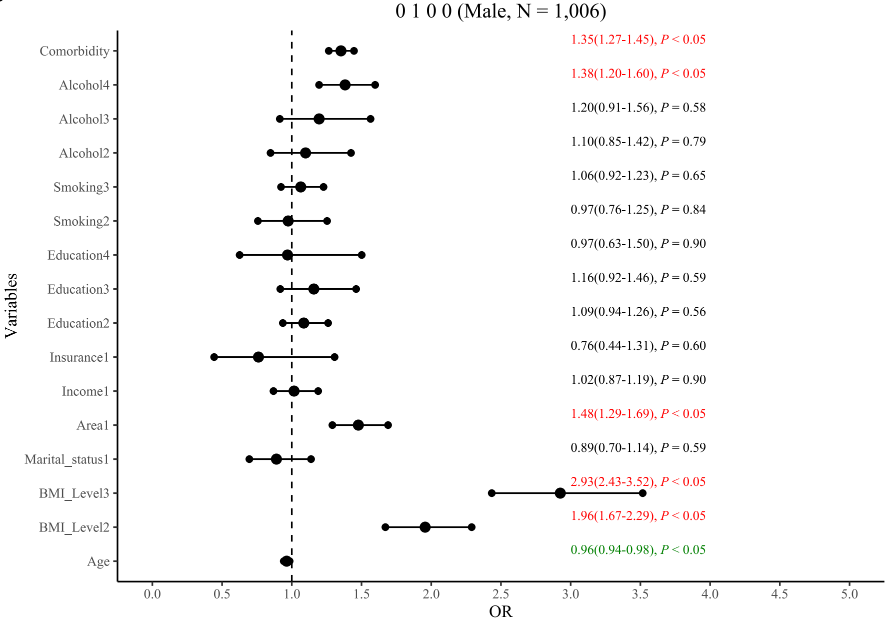

Supplemental Figure.4.1C

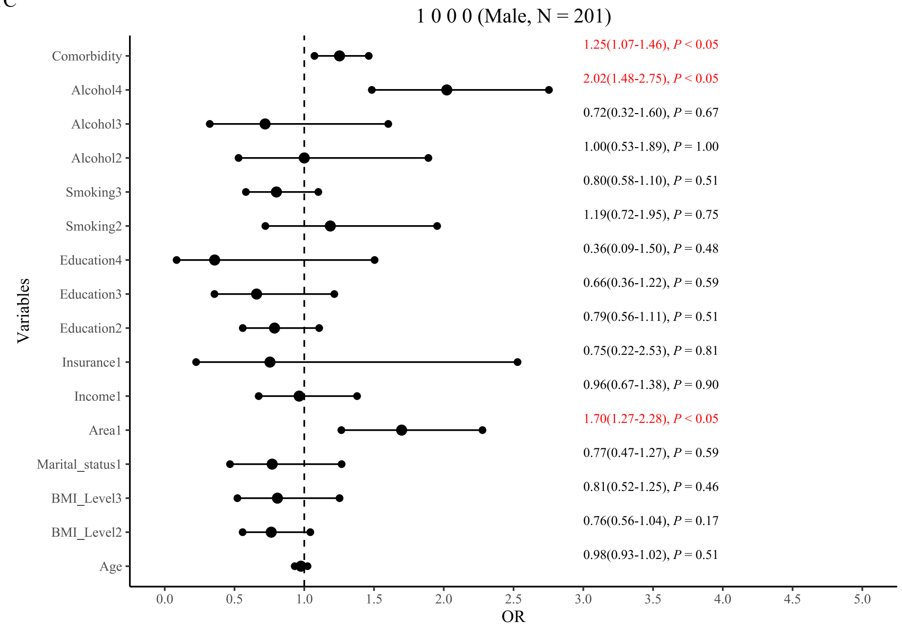

Supplemental Figure.4.1D

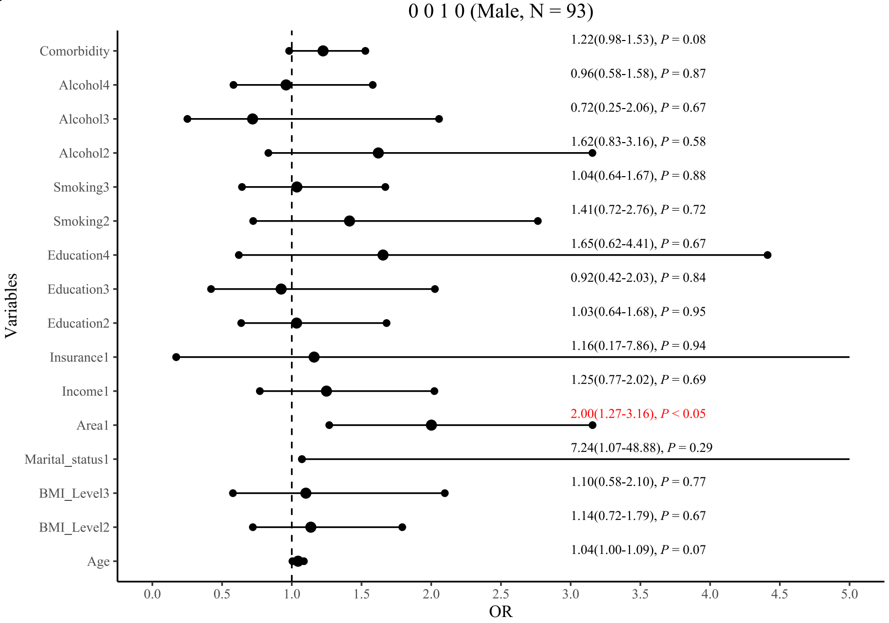

Supplemental Figure.4.2 Single Dyslipidemia Patterns and Associated Factors among Females

Supplemental Figure.4.2A

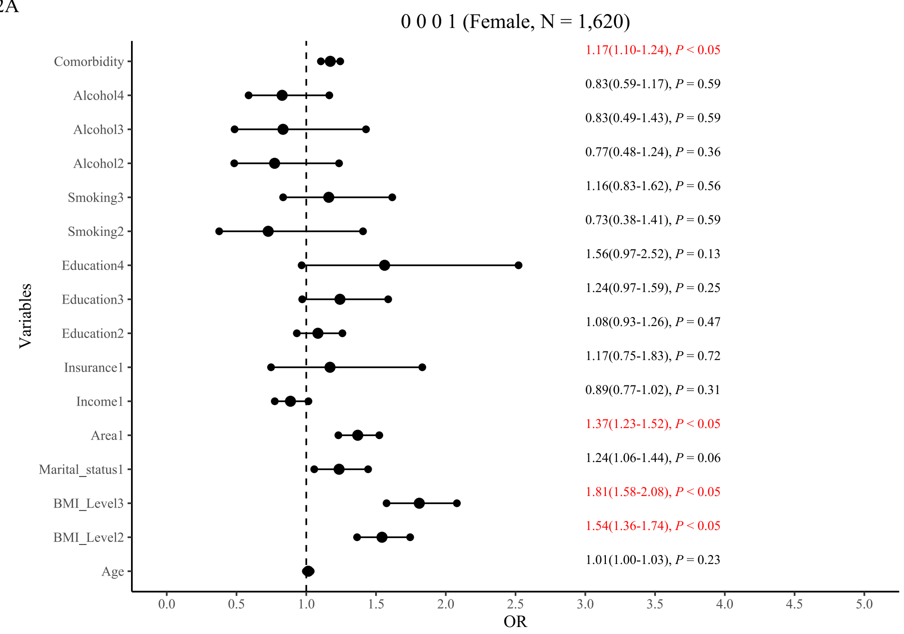

Supplemental Figure.4.2B

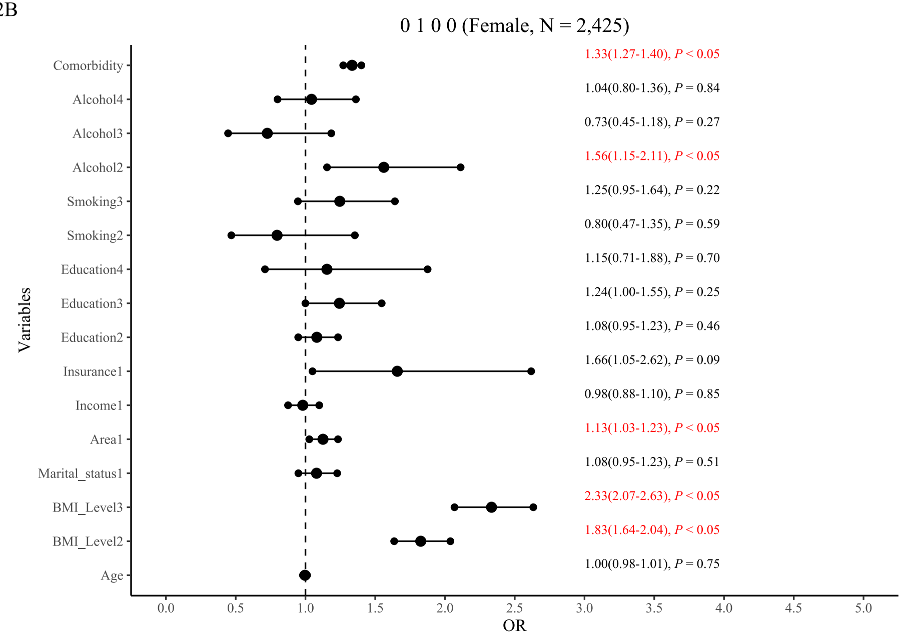

Supplemental Figure.4.2C

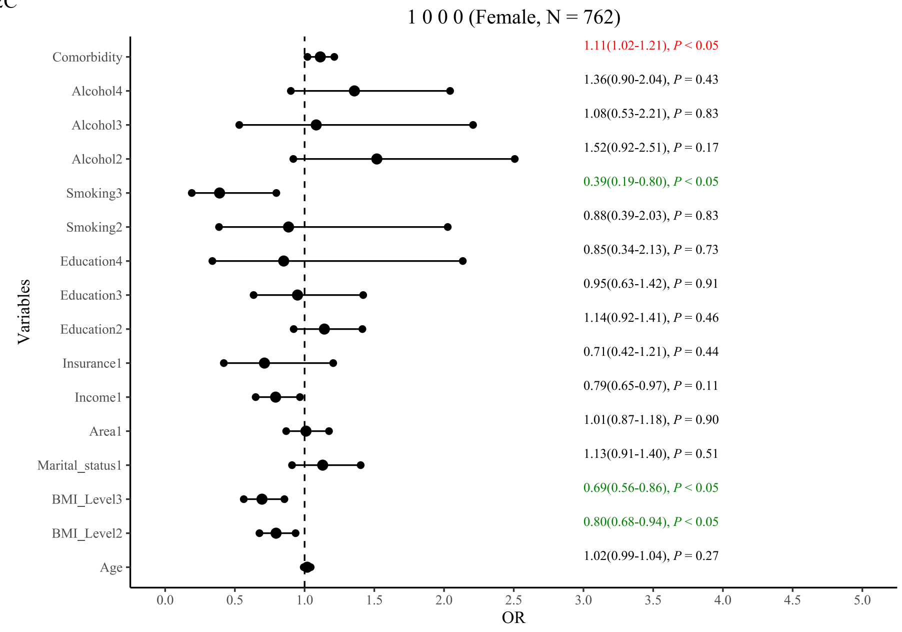

Supplemental Figure.4.2D

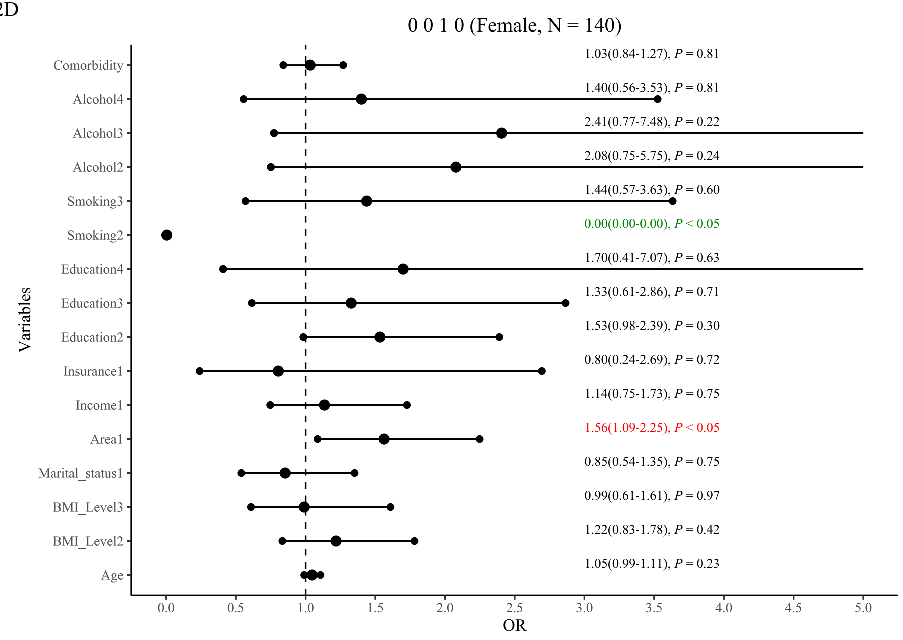

Supplemental Figure.5.1 Mixed & Complex Dyslipidemia Patterns and Associated Factors among Males

Supplemental Figure.5.1A

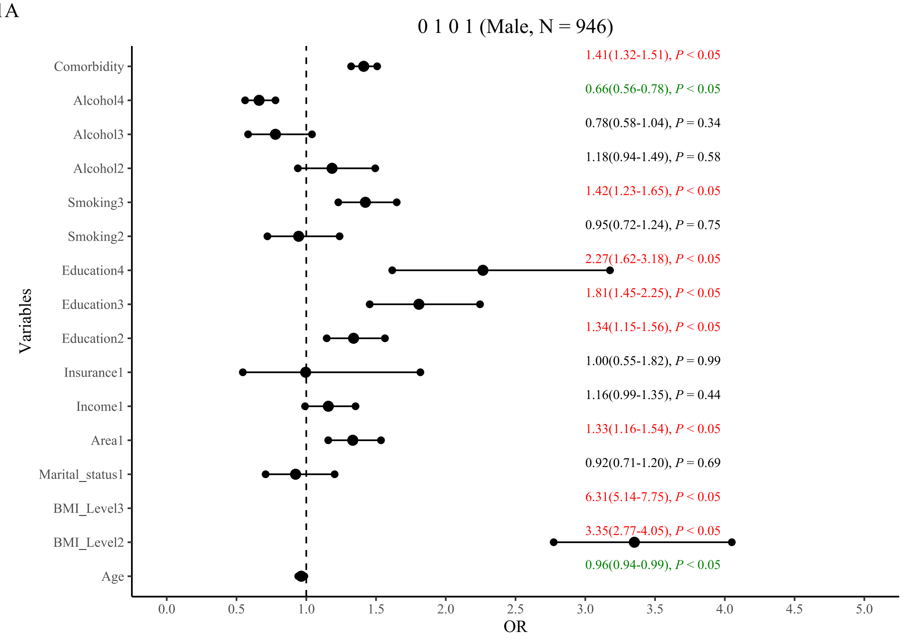

Supplemental Figure.5.1B

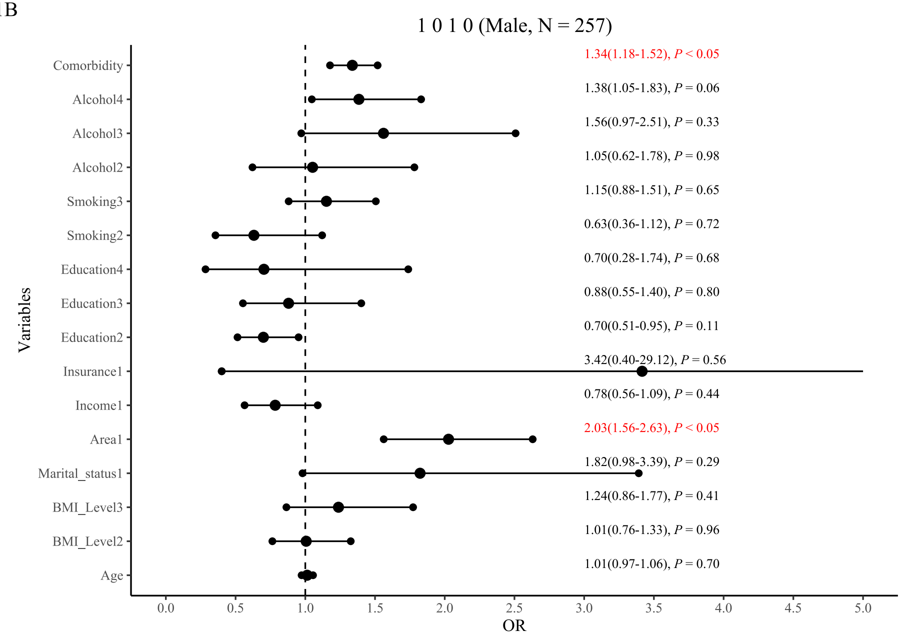

Supplemental Figure.5.1C

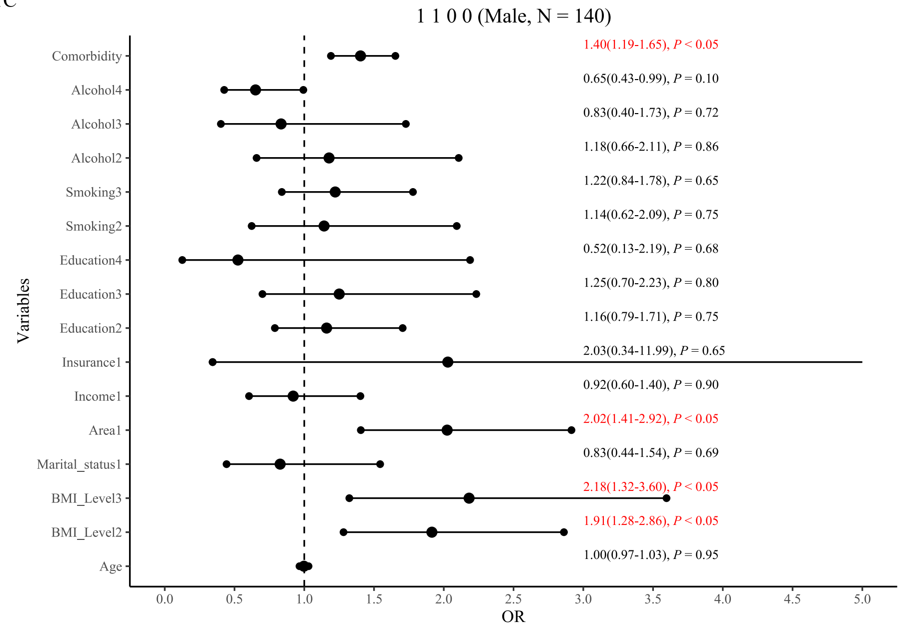

Supplemental Figure.5.1D

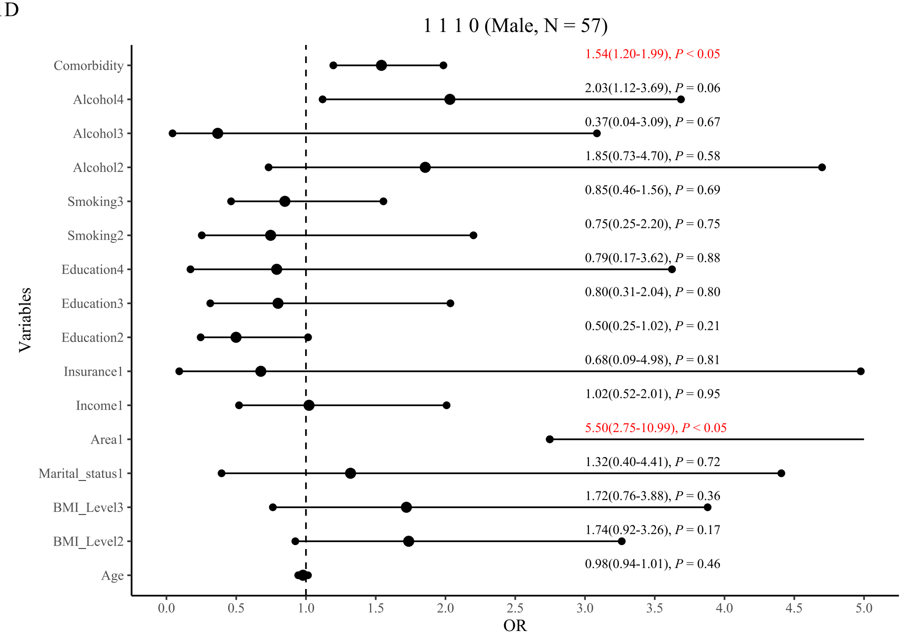

Supplemental Figure.5.2 Mixed & Complex Dyslipidemia Patterns and Associated Factors among Females

Supplemental Figure.5.2A

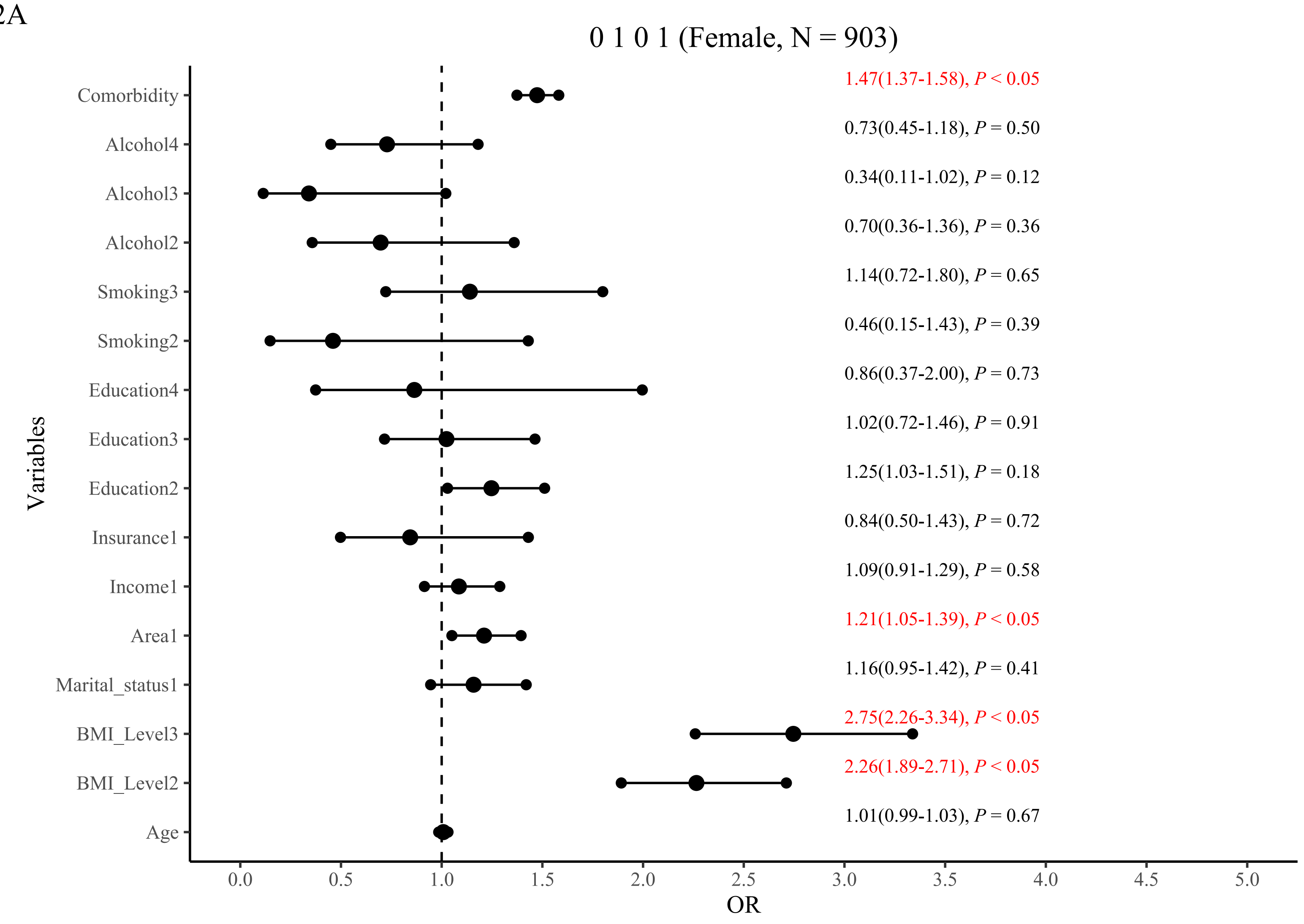

Supplemental Figure.5.2B

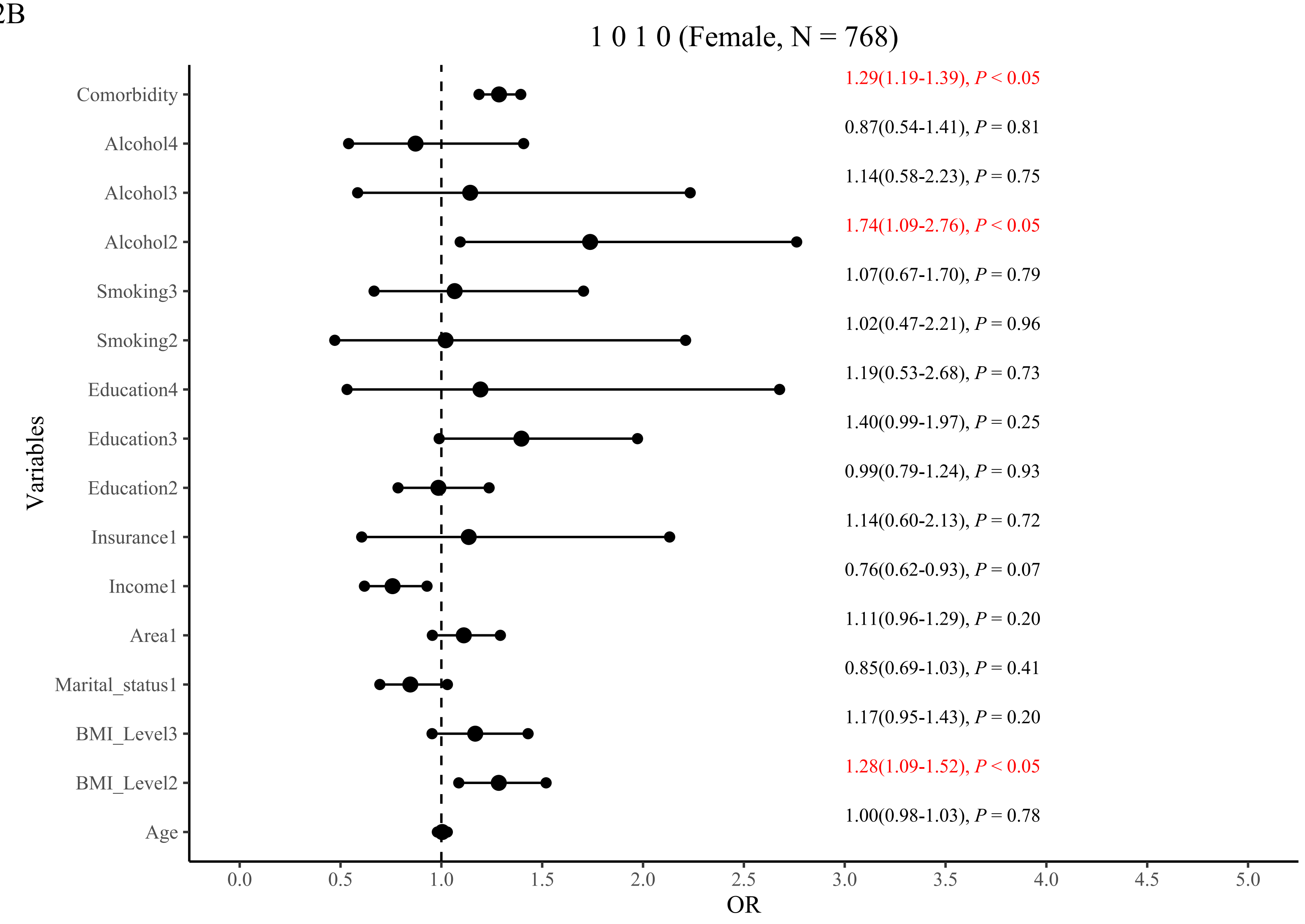

Supplemental Figure.5.2C

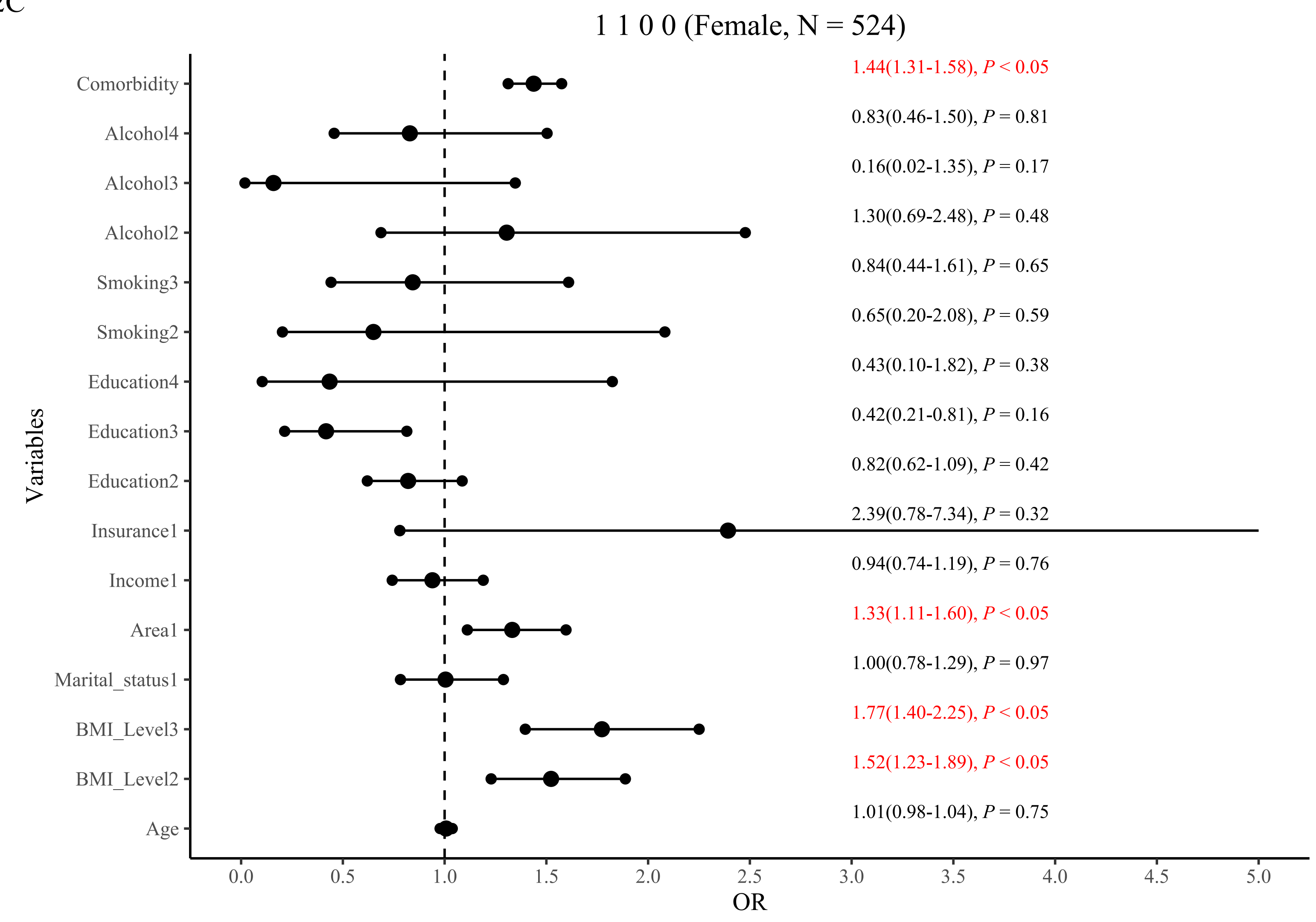

Supplemental Figure.5.2D

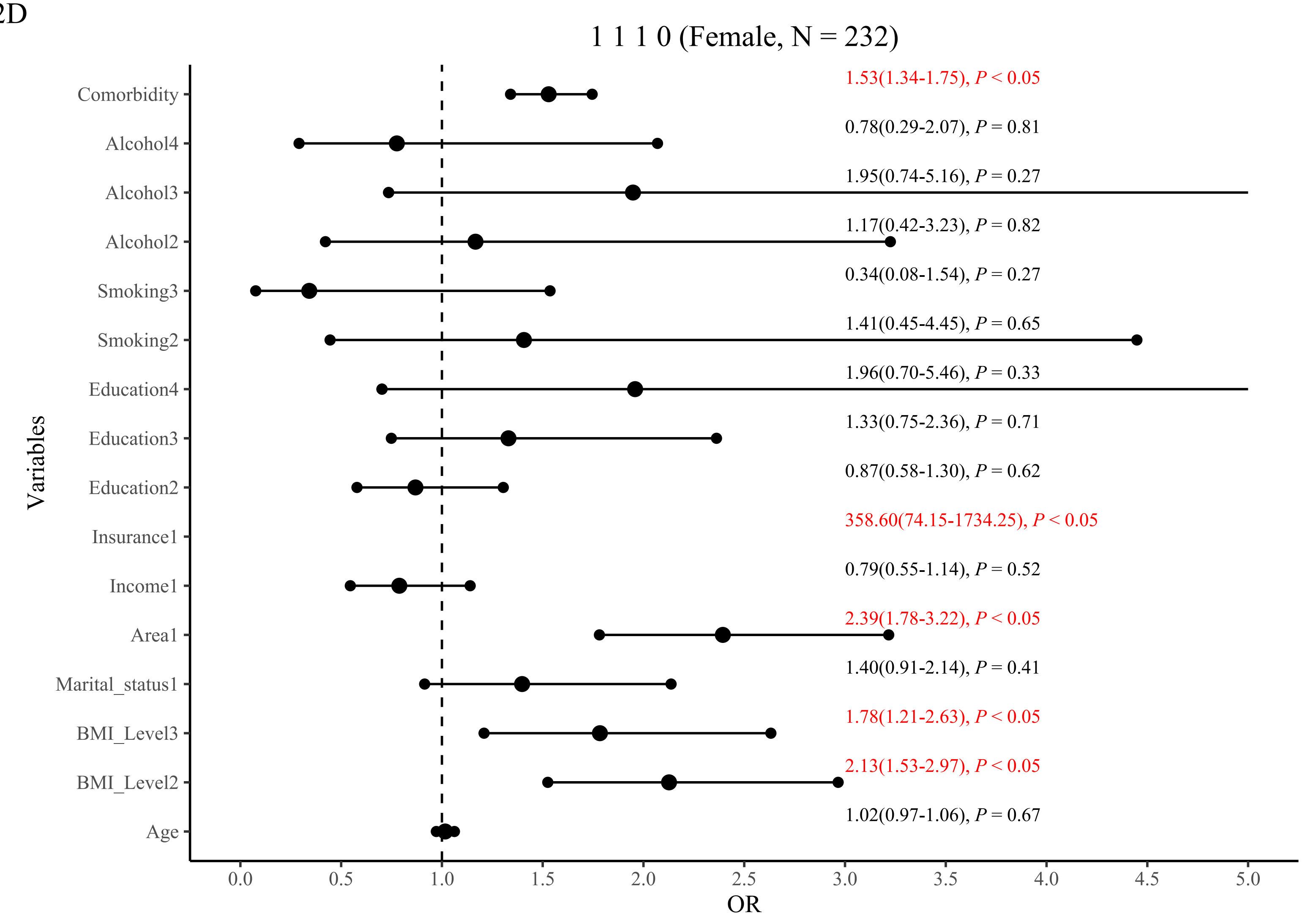

Supplement: Supplementary file 1 [file Datasheet1.pdf]
